# Familial neonatal seizures caused by the Kv7.3 selectivity filter mutation T313I

**DOI:** 10.1002/epi4.12438

**Published:** 2020-10-17

**Authors:** Jasmine Maghera, Jingru Li, Shawn M. Lamothe, Marvin Braun, Juan P. Appendino, P. Y. Billie Au, Harley T. Kurata

**Affiliations:** ^1^ Department of Pharmacology Alberta Diabetes Institute University of Alberta Edmonton AB Canada; ^2^ Division of Child Neurology Department of Pediatrics Weill Cornell Medicine New York NY USA; ^3^ Section of Neurology Department of Pediatrics Cumming School of Medicine University of Calgary, and Alberta Children’s Hospital Calgary AB Canada; ^4^ Department of Medical Genetics Cumming School of Medicine Alberta Children’s Hospital Research Institute University of Calgary Calgary AB Canada

**Keywords:** epilepsy, KCNQ2, KCNQ3, Kv7, neonatal, potassium channel, seizure

## Abstract

**Objective:**

A spectrum of seizure disorders is linked to mutations in Kv7.2 and Kv7.3 channels. Linking functional effects of identified mutations to their clinical presentation requires ongoing characterization of newly identified variants. In this study, we identified and functionally characterized a previously unreported mutation in the selectivity filter of Kv7.3.

**Methods:**

Next‐generation sequencing was used to identify the Kv7.3[T313I] mutation in a family affected by neonatal seizures. Electrophysiological approaches were used to characterize the functional effects of this mutation on ion channels expressed in *Xenopus laevis* oocytes.

**Results:**

Substitution of residue 313 from threonine to isoleucine (Kv7.3[T313I]) likely disrupts a critical intersubunit hydrogen bond. Characterization of the mutation in homomeric Kv7.3 channels demonstrated a total loss of channel function. Assembly in heteromeric channels (with Kv7.2) leads to modest suppression of total current when expressed in *Xenopus laevis* oocytes. Using a Kv7 activator with distinct effects on homomeric Kv7.2 vs heteromeric Kv7.2/Kv7.3 channels, we demonstrated that assembly of Kv7.2 and Kv7.3[T313I] generates functional channels.

**Significance:**

Biophysical and clinical effects of the T313I mutation are consistent with Kv7.3 mutations previously identified in cases of pharmacoresponsive self‐limiting neonatal epilepsy. These findings expand our description of functionally characterized Kv7 channel variants and report new methods to distinguish molecular mechanisms of channel mutations.

**Key Points**

- The Kv7.3[T313I] mutation was identified in a family with heritable pharmacoresponsive self‐limiting neonatal seizures
- Functional characterization revealed that Kv7.3[T313I] mutant subunits assemble with Kv.7.2 and suppress currents, but have little effect on gating parameters
- These findings validate Kv7.3[T313I] as a pathogenic mutation in neonatal epilepsy

## 1 INTRODUCTION

Mutations of the KCNQ2 and KCNQ3 voltage‐gated potassium channel genes have been implicated in the pathophysiology of a spectrum of seizure disorders, ranging from pharmacoresponsive self‐limiting neonatal epilepsy to severe epileptic encephalopathy.^1^, ^2^, ^3^, ^4^, ^5^, ^6^, ^7^ The milder end of this spectrum has been historically referred to as BFNE (Benign Familial Neonatal Epilepsy) and is characterized by seizures that begin in the first few postnatal days, which are typically pharmacoresponsive and self‐limiting within a few months and rarely to a few years, and do not interfere with structural or cognitive development.^8^, ^9^ The term BFNE has been discouraged in recent position statements because the term “Benign” may minimize the impact of the disease on patients and caregivers.^10^ One issue often encountered clinically is how to best predict long‐term outcomes and approach management for a neonate with new‐onset seizures. Increased access to genetic sequencing has allowed clinicians to rapidly identify genetic forms of epilepsy, but there is often still insufficient information for an accurate prediction of phenotype from genotype, particularly for mutations in KCNQ2 and KCNQ3, which can present with a wide range of epileptic severity and may have overlapping features.^11^, ^12^

KCNQ2 and KCNQ3 gene products, Kv7.2 and Kv7.3, form heterotetrameric potassium channels that encode the neuronal M‐current,^13^ which plays a modulatory role on threshold properties and repetitive burst firing in response to excitatory stimuli.^14^, ^15^, ^16^, ^17^ Heteromeric assembly of Kv7.2 and Kv7.3 subunits enhances expression of channels in heterologous systems by roughly 10‐fold relative to homomeric channels of either subtype alone,^18^ and so dysfunction in either subunit can be linked to a pathological outcome. In general, Kv7.2 is more frequently implicated in both self‐limiting seizures and epileptic encephalopathy phenotypes. Pathogenic variants are less frequently identified in Kv7.3.^19^

**Figure 1:**
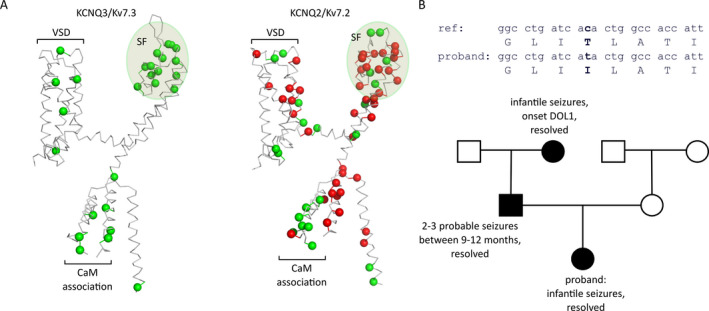
Inheritance of Kv7.2 and Kv7.3 mutations associated with epilepsy. A, Mutations in Kv7.2 or Kv7.3 associated with a documented case of epilepsy (compiled from ClinVar or RIKEE databases) are highlighted on molecular models of each channel. Mutations are color coded based on severity (green = BFNE, red = epileptic encephalopathy or other severe outcomes). Mutations that do not map to structural elements defined in the KCNQ1 cryo‐EM structure have been omitted (VSD, voltage‐sensing domain, SF, selectivity filter, CaM, Calmodulin). B, Pattern of inheritance of a neonatal seizure phenotype in a family carrying the Kv7.3[T313I] mutation. Upper, sequence alignment of the reference KCNQ3 gene and Kv7.3 protein in relation to the proband. The identified mutation [T313I] is highlighted in bold type. Lower, pedigree for the family characterized in our study with filled symbols indicating affected individuals

Kv7.3 mutations have been reported throughout the channel sequence, including C‐terminal domains associated with calmodulin or PIP2 association and the voltage‐sensing domain. This is highlighted in Figure 1A, illustrating the location of pathogenic point mutations of Kv7.3 that are reported in clinVar, on the structural template of a recently reported Kv7.1 cryo‐EM structure.^20^ There is a cluster of validated disease‐linked mutations in the Kv7.3 selectivity filter region, which underlies ion selectivity and permeation.^5^, ^21^, ^22^, ^23^, ^24^, ^25^ The geometry and stability of the selectivity filter are determined by several intra‐ and intersubunit interactions that are widely conserved among K^+^ channels.^26^, ^27^, ^28^ In some channel types prone to selectivity filter mediated inactivation, the disruption of these bonds leads to pronounced alterations of the kinetics of inactivation or a total loss of channel conductance.^29^, ^30^, ^31^, ^32^ In contrast, M‐channels exhibit no apparent inactivation, and selectivity filter mutations can lead to a range of effects on overall channel function.^13^, ^16^, ^33^

In this study, we report a family with a pattern of inheritance and symptoms indicating a diagnosis of pharmacoresponsive self‐limiting seizures (BFNE). The proband was identified to have a KCNQ3 variant, p.Thr313Ile, which is a previously unreported selectivity filter mutation in Kv7.3. A mutation has been reported in Kv7.2 channels at a homologous position (T274M), associated with a severe clinical outcome including profound global developmental delay, motor dysfunction, and remitting pharmacoresistant seizures.^7^, ^33^, ^34^, ^35^ We undertook the characterization of the electrophysiological effects of the T313I mutation in Kv7.3 homomeric and Kv7.2/Kv7.3 heteromeric channels, to describe in more detail the potential effects of selectivity filter mutations in this channel family. We also developed an assay using a subtype‐specific Kv7.2 activator (ICA‐069673) as a “fingerprinting” tool to distinguish functional Kv7.2/Kv7.3 heteromeric channels from Kv7.2 homomers.^36^, ^37^, ^38^ Our findings highlight variable outcomes of mutations in this critical selectivity filter position on the overall function of Kv7.2/Kv7.3 heteromeric channels. We find that Kv7.2/Kv7.3 heteromers are relatively tolerant of the Kv7.3[T313I] mutation, and this may underlie the mild disease phenotype, in contrast to severe outcomes arising from Kv7.2[T274M].^33^

## 2 MATERIALS AND METHODS

### 2.1 Molecular biology and plasmid construction

The KCNQ3[T313I][A315T] and KCNQ3[T313I] mutation were generated by site‐directed mutagenesis in pSRC5 (gift from Dr. M. Taglialatela, University of Naples, Italy, and Dr. T. Jentsch, Max‐Delbrück‐Centrum für Molekulare Medizin, Germany) and pTLN plasmids encoding Kv7.3.

Primers used for mutagenesis for the KCNQ3[T313I] construct were as follows (together with their reverse complement primer):

KCNQ3[T313I]: 5′‐GCCTGATCATACTGGCCACCATTGGC‐3′

KCNQ3[T313I]: 5′‐GCCTGATCATGCTGGCCACCATTGGC‐3′

KCNQ3[A315T][T131I]: 5′‐GCCTGATCATACTGACCACCATTGGC‐3′

KCNQ2[T274I]: 5′‐GGGGCCTGATCATCCTGACCACCATTG‐3′

KCNQ2[T274M]: 5′‐GGGGCCTGATCATGCTGACCACCATTG‐3′

### 2.2 Two‐electrode voltage‐clamp expression and recording

Complementary RNA was transcribed from cDNA to express the monomeric KCNQ3 constructs in *Xenopus laevis* oocytes using the mMessage mMachine kit (Ambion). Constructs expressed in pSRC5 (KCNQ3[T313I][A315T] or (KCNQ3[A315T])) were linearized with ApaL1 and transcribed using a T7 primer. Constructs in pTLN (KCNQ2, KCNQ3, and KCNQ3[T313I]) were linearized with HpaI and transcribed using an SP6 primer. Oocyte preparation and RNA injection were performed as described previously,^39^ under the approval of the University of Alberta Animal Care protocol AUP00001752. After injection, oocytes were incubated for 48 hours at 18°C before electrophysiological recording. Voltage‐clamp recordings were obtained in modified Ringer’s solution (in mmol/L): 116 NaCl, 2 KCl, 1 MgCl2, 0.5 CaCl2, and 5 HEPES, pH adjusted to 7.4 with NaOH, using an OC‐725C voltage clamp (Warner). Microelectrodes were backfilled with 3 mol/L KCl to obtain resistance between 0.1 and 1 MΩ. Data were processed using a Digidata 1440A acquisition system controlled by pClamp 10 software. For experiments using ICA‐069673, oocytes were incubated in 100 μmol/L ICA‐069673 in modified Ringer's solution for 3‐4 minutes before recording.

### 2.3 Data analysis and statistics

Statistical tests used throughout the manuscript are described in the corresponding figure legends. Gating parameters describing voltage dependence of channel activation were determined by fitting with a standard single‐component Boltzmann equation of the form:

$$G=\frac{1}{\left(1+e^{‐(V‐V_{1/2})/k}\right)}$$

where *V*_1/2_ is the voltage where channels exhibit half‐maximal activation, and *k* is a slope factor reflecting the voltage range over which an *e*‐fold change in open probability (Po) is observed. The extent of tail current deactivation in the presence of ICA‐069673 was inferred by the ratio of the magnitude of instantaneous: peak current after the repolarization interval, rather than fitting tail current kinetics directly (these are often too slow to generate a meaningful fit).

### 2.4 DNA sequencing

Patient samples were sequenced clinically by an external provider (CeGAT GmbH, Tubingen, Germany). The proband was initially investigated using a metabolic/mitochondrial epilepsy panel in order to rule out potentially treatable metabolic causes. Given the family history, some individual genes associated with pharmacoresponsive self‐limiting seizures were also included in addition to the standard panel. The genes examined as part of the standard metabolic epilepsy clinical panel were as follows: AARS2, ABAT, ABCC8, ACY1, ADCK3, ADK, ADSL, ALDH5A1, ALDH7A1, AMT, ATIC, AUH, BCKDHA, BCKDHB, BCKDK, BCS1L, BTD, C10ORF2, CAD, CARS2, CNNM2, COQ4, COX8A, CPT1A, CPT2, D2HGDH, DARS2, DBT, DHFR, DLD, DNM1L, DPYD, EARS2, ETFA, ETFB, ETFDH, ETHE1, FARS2, FOLR1, FOXRED1, GAMT, GATM, GCDH, GCH1, GCK, GCSH, GFM1, GLDC, GLUD1, GLUL, GPHN, HADH, HLCS, HPD, 1DH2, INSR, ITPA, IVD, KCNJ11, L2HGDH, LIAS, MDH2, MLYCD, MMACHC, MOCS1, MOCS2, MT‐ATP6, MT‐TK, MT‐TL1, MTHFR, NARS2, NDUFA1, PC, PCBD1, PCCA, PCCB, PDHA1, PDHX, PDSS2, PET100, PHGDH, PNPO, POLG, PROSC, PSAT1, PSPH, PTS, QDPR, SDHA, SLC16A1, SLC19A3, SLC1A2, SLC25A1, SLC2A1, SLC46A1, SLC6A8, SLC6A9, SUOX, SURF1, VARS2. In addition, KCNQ2, KCNQ3, and PRRT2 sequences were added for analysis based on a suspicion of a BFNE diagnosis. The T313I mutation was identified in the proband, and in silico predictors suggested the variant was damaging. The T313I variant was classified as a VUS because this position had not been previously associated with disease and was not present in the EVS, gnomAD, or RIKEE databases.^40^ Targeted testing was then used to determine the presence of the variant in other family members. Targeted testing confirmed the presence of the variant in the father and paternal grandmother who also have a history of early seizures and absence of the variant in the mother who has no history of seizure.

## 3 RESULTS

### 3.1 Proband features

The proband was a term infant female born after an unremarkable pregnancy and uncomplicated vaginal delivery. At 4 days of age, she began having spells described as “not breathing properly” while “holding her arms and legs out (flexed or extended) with a mild tremor and at times hyperextension of her back”. The events increased in frequency, beginning to cluster, and she was admitted to the hospital for workup. Seizures were suspected and she was loaded with 20 mg/kg of IV levetiracetam and continued with 10 mg/kg bid of maintenance. Video‐EEG monitoring was initiated. Six discrete, self‐limited electroclinical seizures were recorded in sleep and wakefulness in the first 24 hours of recording. The seizures lasted between 43 and 69 seconds (mean 54 seconds) and while the semiology varied slightly, the typical pattern entailed three clearly discernible stages: (a) wide eye opening, with occasional gaze deviation to the left, (b) tonic posturing of limbs and torso (typically left arm extension followed by arching of back and flection or extension of legs), and (c) clonic movements of bilateral arms and legs until offset. The ictal EEG correlate consisted of symmetric background attenuation with overriding low amplitude fast beta frequencies for ~15 seconds (tonic phase) followed by bilateral high amplitude spike‐sharp delta‐theta frequencies in a crescendo‐decrescendo manner (clonic phase) until offset. Interictally, the EEG showed bilateral symmetric and continuous background activity with mixture of delta‐theta frequencies and rare (<1/min) non‐specific negative sharp waves with independent maximum amplitude over T7, T8, C3, C4, or Cz. Normal wake‐sleep cycling was also seen.

A neurological examination revealed a normal non‐dysmorphic infant without focal neurological deficits. Brain MRI showed normal anatomy. On review of the pregnancy history, the patient’s mother was healthy, with a history of 3 previous early pregnancy losses. There was hyperemesis in the first trimester but pregnancy was otherwise unremarkable with no drug or alcohol exposures, and the patient was delivered at 40 weeks gestation. On review of the family history, the infant’s father had a history of seizures in early infancy (9‐12 months) that were self‐limiting. He also has attention deficit hyperactivity disorder but normal cognition. The patient’s paternal grandmother had seizures in infancy, which were also self‐limiting and normal cognition. There is also a paternal cousin with epilepsy. There was no history of seizure in the patient’s mother or maternal relatives (Figure 1B).

With the continuation of her seizures, the maintenance dose of IV levetiracetam was increased to 15 mg/kg bid without effect. She was then loaded with 10 mg/kg of IV phenobarbital and the seizures stopped. Given the efficacy of phenobarbital, her levetiracetam was discontinued and she was kept on maintenance phenobarbital (2.5 mg/kg bid). Due to the initial uncertainty of the etiology of this patient’s seizures, effective control only after initiating phenobarbital, and that the patient’s father had sporadic seizures in infancy (9–12 months), a maintenance dose was kept for safety until a clear etiology was found. The patient remained event‐free and a repeat EEG at 6 months of age was normal. With the normal EEG, lack of clinical events, and normal development, phenobarbital was weaned at 8 months of age. Seizures had remained after levetiracetam initiation; therefore, seizure cessation was suspected to be due to phenobarbital effects. Nevertheless, one cannot rule out spontaneous seizure remission. Seizures did not relapse despite being off anti‐seizure therapy, and she remains seizure‐free for more than 3 years. Development also remained normal in all domains, suggesting a clinical presentation consistent with self‐limiting pharmacoresponsive seizures.

Metabolic investigations for this patient were unremarkable. Clinical genetic testing had been initiated for this patient at seizure onset. Array CGH (60K) was negative. A 114 gene epilepsy panel through the clinical commercial laboratory CeGAT (see Materials and Methods) identified a variant of uncertain significance in KCNQ3, c.938C>T, p.Thr313Ile (NM_004519) (Figure 1B). This variant was not identified in the patient’s mother, but was present in the patient’s father and paternal grandmother (Figure 1B). This variant is absent from gnomAD and was not previously reported in ClinVar, EVS, or RIKEE databases.^40^, ^41^

### 3.2 KCNQ3[T313I] mutation eliminates homomeric Kv7.3 function

**Figure 2:**
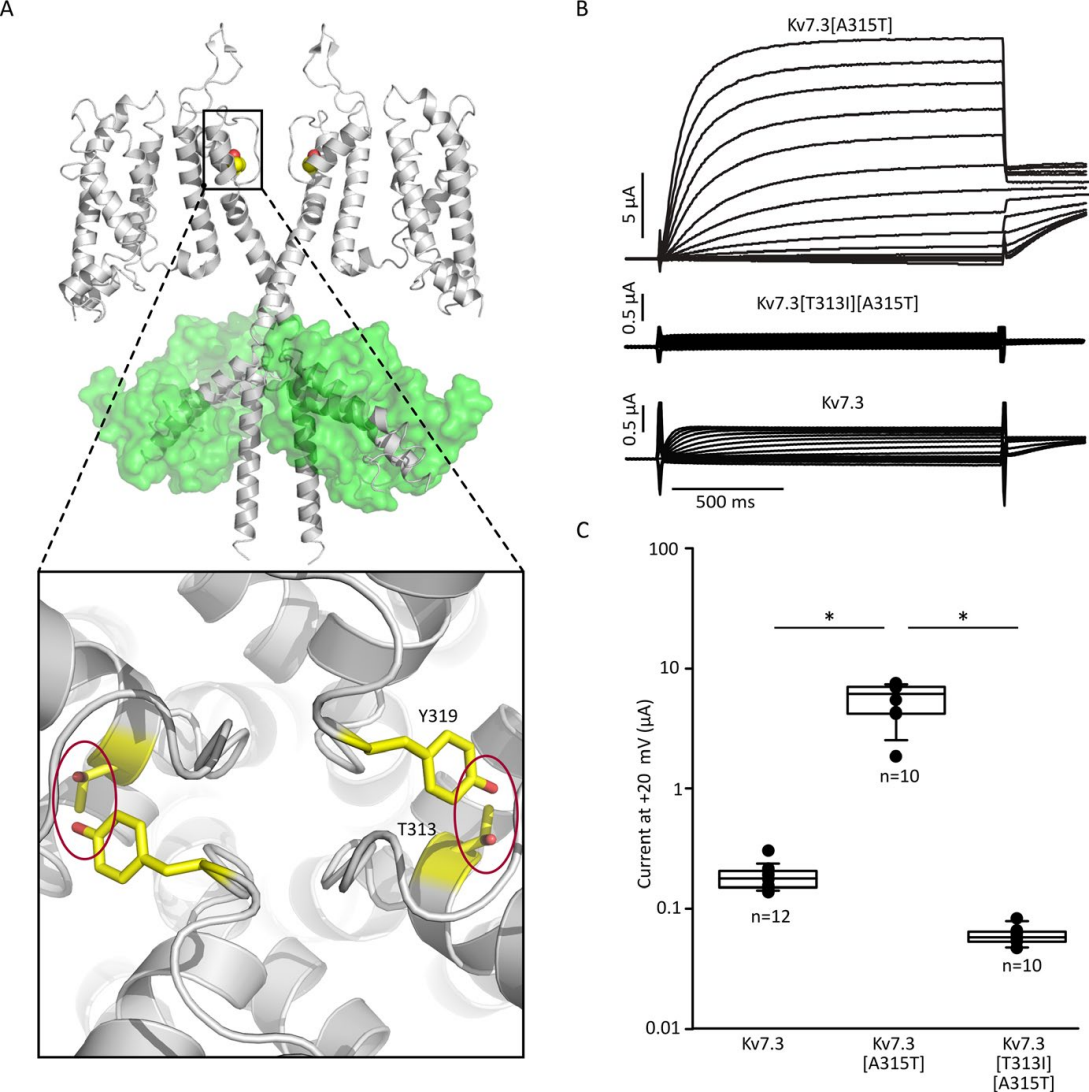
Kv7.3[T313I] abolishes Kv7.3 function. A, Molecular model of essential intersubunit hydrogen bond between conserved residues T313 and Y319 located in the selectivity signature sequence. B, Two‐electrode voltage‐clamp recordings from *Xenopus laevis* oocytes expressing Kv7.3[A315T] and Kv7.3[A315T][T313I]. Oocytes were held at −80 mV and depolarized for 1.5s to voltages between −140 mV and +40 mV (in 10‐mV steps) followed by repolarization to −20 mV test pulse. Current amplitudes at +20 mV of Kv7.3 [A315T] and Kv7.3[A315T] [T313I] were compared using a student’s t test (* indicates *P*<0.05 relative to A315T alone)

We were intrigued by both the biophysical and physiological consequences of the T313I mutation. The T313 position is one of several positions identified in patients with self‐limiting neonatal seizures, clustered in the region of the selectivity filter (Figure 1A). Due to the critical functional importance of this region, variants in the vicinity of the selectivity filter are often linked to disease (D305, A306, W308, W309, G310, I317, Y319, G320 are listed in ClinVar and linked to BFNE). Despite the essential role of the selectivity filter, the pathology arising from these mutants is relatively mild and self‐resolving. A previous study of an analogous mutation in the widely studied *Shaker* potassium channel, a model system for understanding many basic principles of voltage‐dependent ion channel function, highlighted the importance of this particular site in maintaining the stability of the selectivity filter.^29^ Kv7.3 position T313 is analogous to *Shaker* residue T439, which is predicted to form an intersubunit hydrogen bond with the selectivity filter residue Y445 (G**Y**G), highlighted in Figure 2A (2 of 4 potential H‐bonds are shown). In *Shaker* channels, disruption of this H‐bond in even a single subunit caused a profound suppression of overall channel current and acceleration of inactivation by ~100‐fold.^29^ These powerful effects were attributed to the intersubunit nature of the interaction, leading to a propagated effect of a mutation from a single subunit (Figure 2A). Similarly, the Kv7.2[T274M] mutation of an analogous position in Kv7.2 has been associated with severe epileptic encephalopathy and dominant negative effects when co‐expressed with Kv7.3.^33^ However, the physiological outcomes in this family carrying the Kv7.3[T313I] mutation were much less severe. We initially characterized the effects of the T313I mutation in homomeric Kv7.3, using the A315T mutation to enable expression of functional homotetramers.^42^, ^43^ Consistent with prior studies in *Shaker* channels, we observed that the T313I mutation virtually abolished functional current relative to Kv7.3[A315T] channels (Figure 2B,C).

### 3.3 Kv7.3[T313I] attenuates currents in Kv7.2/Kv7.3 heteromers

**Figure 3:**
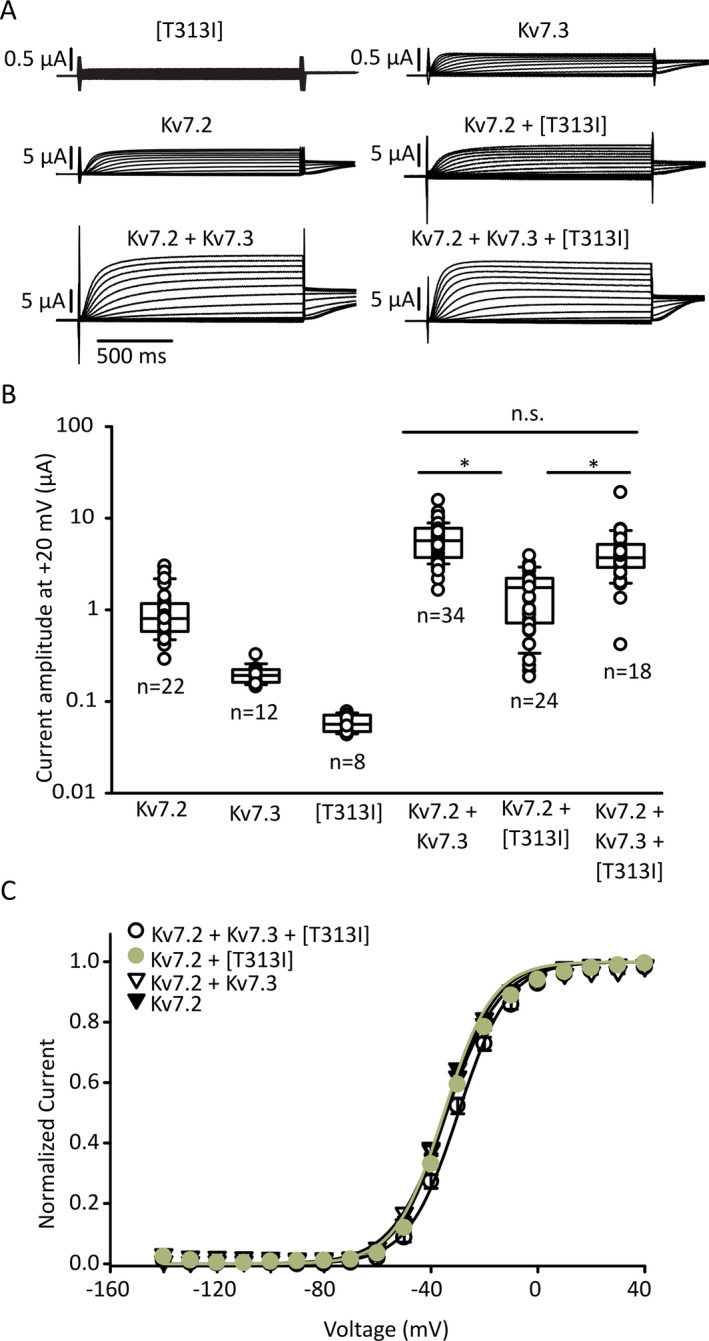
Co‐expression with Kv7.2 and Kv7.3[T313I] reduces heteromeric channel function with no effect on gating. A, Two‐electrode voltage‐clamp sample traces from oocytes expressing various combinations of Kv7.2 and Kv7.3 (injected with a total of 50 ng of mRNA per group). The voltage step protocol is the same as Figure 2B. B, Current amplitudes after a 1.5 s + 20 mV voltage step. Current magnitudes were compared using one‐way ANOVA, followed by Tukey’s post hoc test (* indicates *P* < 0.05). C, Conductance‐voltage relationships were collected using the protocol in panel (A), using tail current magnitudes (−20 mV) to assess the extent of channel opening during the conditioning step. Fitted gating parameters were (mean ± S.E.M.): for Kv7.2 + Kv7.3 +[T313I], k = 9.4 ± 0.3 mV, V_1/2_ = −30 ± 1 mV; for Kv7.2 + [T313I], k = 9.1 ± 0.3 mV, V_1/2_ = −35.1 ± 0.5 mV; for Kv7.2 + Kv7.3, k = 10.2 ± 0.3 mV, V_1/2_ = −34.2 ± 0.3 mV; for Kv7.2 homomers, k = 9.2 ± 0.2 mV, V_1/2_ = −34.2 ± 0.6 mV. No significant differences in gating parameters were detected

Although this profound effect of Kv7.3[T313I] was consistent with previous reports in *Shaker* channels and Kv7.2, it does not address the effects of the mutation in heteromeric Kv7.2/Kv7.3 channels that predominate *in vivo*.^19^, ^44^ To investigate the effects of the T313I mutation in heteromeric channels, we co‐expressed different ratios of Kv7.2, Kv7.3, and Kv7.3[T313I] to simulate different heteromeric assemblies that may occur *in vivo*. Wild‐type Kv7.2/Kv7.3 heteromeric channels (1:1 mRNA injection ratio) generated larger currents relative to homomeric Kv7.2 channels. When Kv7.2 and Kv7.3[T313I] subunits were injected in a 1:1 ratio, the current was reduced significantly relative to the wild‐type heteromer (Figure 3A,B).

We also tested the mixed expression of WT Kv7.3 and Kv7.3[T313I] channels with Kv7.2, to mimic the heterozygous genotype of the proband (Figure 3A,B). A 1:0.5:0.5 (Kv7.2:Kv7.3:Kv7.3[T313I]) RNA injection ratio did not suppress current magnitude relative to WT Kv7.2/Kv7.3 (Figure 3A,B). However, current magnitudes in these experiments are variable and there may be assembly of various channel stoichiometries even with fixed RNA ratios.^45^ No difference in voltage‐dependent activation was detected in oocytes expressing different ratios of Kv7.2, Kv7.3, and Kv7.3[T313I] (Figure 3C). Although these experiments illustrate modest current suppression by Kv7.3[T313I] in certain subunit combinations, they do not fully reveal the nature of Kv7.3[T313I] mutant subunits on function of heteromeric channels, particularly in light of the severe phenotype arising from the Kv7.2[T274M] mutation.^33^ That is, it was not clear whether currents generated from co‐expression of Kv7.3[T313I] and Kv7.2 were generated by heteromeric channels, or from Kv7.2 homomers that did not assemble with Kv7.3[T313I].

### 3.4 Heteromeric composition determined by ICA‐069673 sensitivity

We recently recognized that the functional effects of the Kv7 activator ICA‐069673 depend strongly on subunit composition.^37^ Kv7.2 homomeric channels exhibit profound deceleration of deactivation in the presence of ICA‐069673, whereas these effects are much weaker in homomeric Kv7.2/Kv7.3 channels (due to weak/absent ICA‐069673 sensitivity of Kv7.3). We used this pharmacological “fingerprint” to investigate whether currents observed in the Kv7.2/Kv7.3[T313I] condition were generated by Kv7.2/Kv7.3[T313I] heteromers or only arise from Kv7.2 homomers (due to strong current suppression by Kv7.3[T313I]).

**Figure 4:**
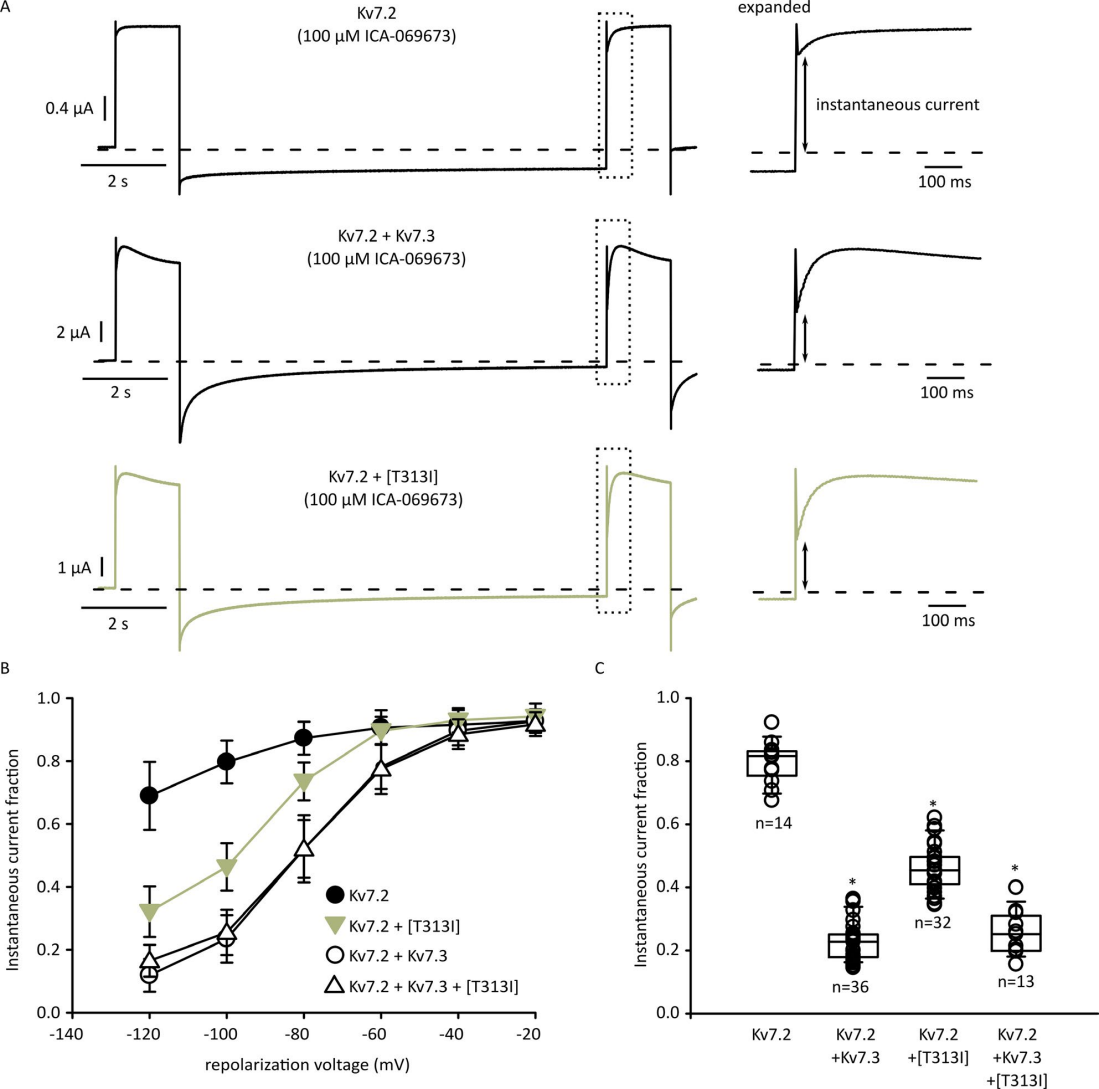
Reduced ICA‐069673 sensitivity of Kv7.2/Kv7.3[T313I] heteromeric channels. A, Example currents of two‐electrode voltage‐clamp recordings. Oocytes were depolarized to +20 mV and repolarized for 12 s in a step‐down manner (−20 mV per sweep), followed by another +20 mV depolarizing pulse to determine instantaneous current at −100 mV. Currents within the dashed box are illustrated on an expanded time scale in the right panels, showing the assessment of instantaneous current levels in different experimental conditions. B, Fractional instantaneous current after incubation with 100 μmol/L ICA‐069673 was measured as indicated by the arrows in panel (A), data are shown as mean ± SEM. C, Fractional instantaneous current (repolarization voltage of −100 mV) for various combinations of Kv7.2, Kv7.3, and Kv7.3[T313I], shown on a cell‐by‐cell basis (Kruskal‐Wallis one‐way ANOVA on ranks, * indicates *P* < 0.05 relative to Kv7.2 homomeric channels)

Using oocytes continuously perfused with a saturating concentration of 100 μmol/L ICA‐069673, we delivered a voltage step protocol that depolarized channels to +20 mV for 1.5 seconds, followed by repolarization to a range of voltages for 12 seconds (Figure 4A). This was followed by a second step to +20 mV to assess the extent of channel closure during the repolarization interval, based on the instantaneous vs activating current fractions elicited by the voltage step (expanded time scales of the second depolarizing step are illustrated in the right hand panels of Figure 4A). Kv7.2 homomeric channels in 100 μmol/L ICA‐069673 are characterized by extremely slow channel closure, resulting in a large instantaneous current fraction, even after repolarization at very negative voltages. In contrast, heteromeric Kv7.2/Kv7.3 channels exhibit much more prominent deactivation during this interval, which results in smaller instantaneous currents (Figure 4A,B). In 100 μmol/L ICA‐069673, Kv7.2 homomeric channels exhibited ~70% instantaneous current after a 12 second repolarization at −120 mV, whereas heteromeric channels generated <20% instantaneous current (Figure 4B,C). Co‐expression of 1:1 Kv7.2:Kv7.3[T313I] channels exhibited an intermediate behavior, but was distinct from WT Kv7.2 homomers. This observation suggests that heteromeric Kv7.2:Kv7.3[T313I] channels retain function, but there is also likely a contribution of homomeric Kv7.2 channels (Figure 4C). Co‐expression to mimic the heterozygous genotype (1:0.5:0.5, Kv7.2:Kv7.3: Kv7.3[T313I]) displayed a similar ICA‐069673 response to the wild‐type heterozygous control (Figure 4C).

### 3.5 Distinct effects of Kv7.2 and Kv7.3 mutations

**Figure 5:**
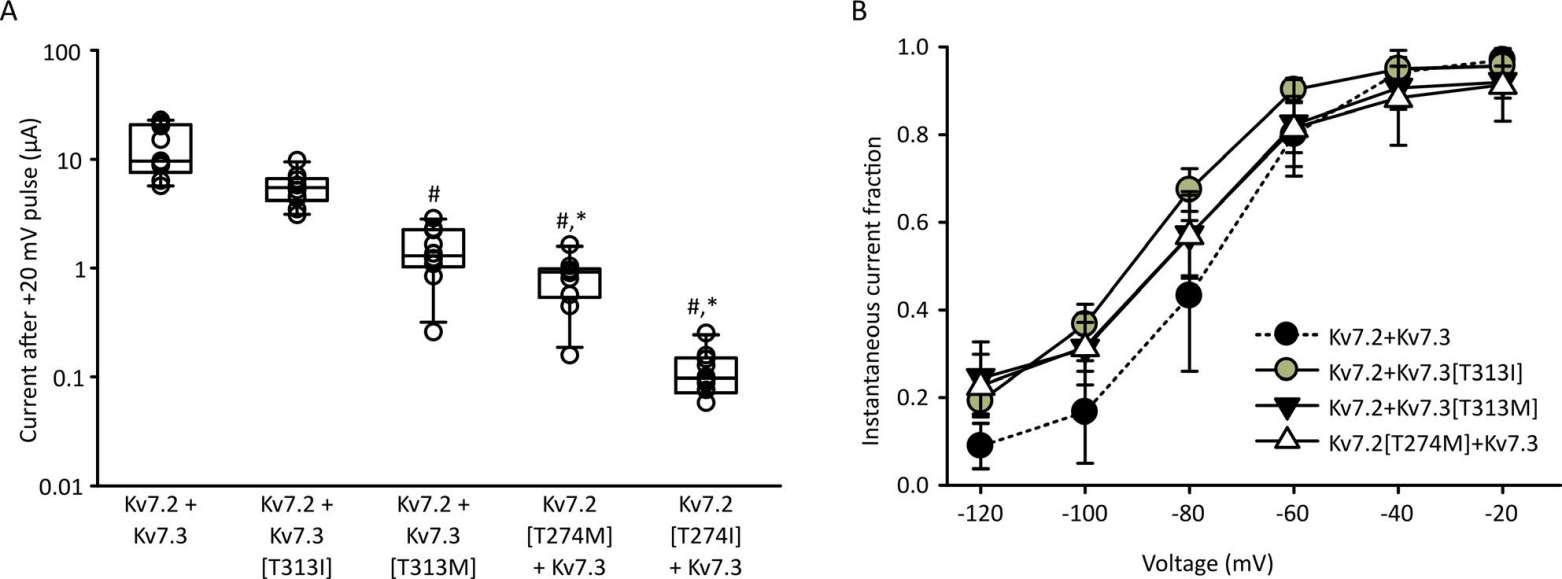
Variable outcomes of homologous selectivity filter mutations in Kv7.2 and Kv7.3. A, Current magnitude was measured at +20 mV in *Xenopus* oocytes injected with Kv7.2 + Kv7.3, or various combinations of channel mutants and their wild‐type counterpart, as indicated. Currents were recording 48‐56 h after injection (n = 10 per condition, one‐way ANOVA on ranks and Tukey post hoc test, # indicates *P* < 0.05 relative to Kv7.2/Kv7.3, * indicates *P* < 0.05 relative to Kv7.2/Kv7.3[T313I]). B, Instantaneous tail current magnitudes were recorded as described in Figure 4 in the presence of 100 μmol/L ICA‐069673, for all possible combinations of injected channels (note that Kv7.3 + Kv7.2[T274I] did not yield current sizes that could be confidently assessed or analyzed)

In order to investigate the functional basis for different clinical outcomes of Kv7.2 and Kv7.3 mutations reported at analogous positions, we also tested the effects of the Kv7.3[T313M] mutation (equivalent to Kv7.2[T274M]) and the Kv7.2[T274I/M] mutations (Figure 5). We measured current amplitude (Figure 5A) and also used the ICA‐069673 response described in Figure 4 to confirm assembly of Kv7.2/Kv7.3 heteromers (Figure 5B). Among these mutant channels, the Kv7.3[T313I] mutant stood out for its comparably mild effects on current magnitude. Co‐expression of Kv7.3[T313M] with Kv7.2 led to a significantly stronger suppression of current relative to Kv7.3[T313I]. Furthermore, co‐expression of Kv7.2[T274M] or Kv7.2[T274I] led to even further suppression of heteromeric Kv7.2/Kv7.3 channel currents. For most of these subunit combinations, we could detect currents resembling Kv7.2/Kv7.3 heteromeric channels based on their weak sensitivity to ICA‐069673 (Figure 5B), although the Kv7.2[T274I] + Kv7.3 combination did not generate sufficient currents in any oocytes to confidently assess any properties of the elicited currents. Overall, these findings confirm that the various Kv7.2 and Kv7.3 mutants can assemble with their WT partner in heteromeric channels, but with very markedly different outcomes on overall channel function.

## 4 DISCUSSION

Determining genotype‐phenotype correlation of mutations in Kv7.2 and Kv7.3 is of significant interest in understanding the prognosis and pathogenesis of affected patients. In addition, characterization of variants is valuable for understanding assembly, function, and regulation of Kv7.2/Kv7.3 heteromeric channels, which play a significant modulatory role in the central nervous system. Delineation of how variants impact channels also informs development of targeted drug therapeutics. In this study, we report a novel mutation in the selectivity filter region of Kv7.3 channels, predicted to form an intersubunit interaction that has been previously shown to strongly disrupt the function of *Shaker* channels, and M‐channels when mutated in Kv7.2.^29^, ^33^ The T313I mutation in Kv7.3 was identified in a family presenting with neonatal self‐resolving pharmacoresponsive epilepsy (frequently referred to as BFNE) and was not previously reported in gnomAD, clinVar, or the KCNQ disease‐specific RIKEE databases. Although this variant was initially classified as a variant of uncertain significance, we feel our findings classify this mutation as pathogenic given its impact on channel function (Figure 3), and due to segregation of the variant with disease in the family.

Previous work in *Shaker* channels demonstrated the contribution of this position to channel function, based on formation of a H‐bond between T439 (equivalent to Kv7.3[T313I]) and Y445 (in the GYG selectivity filter sequence of an adjacent subunit). An important comparison was drawn to a commonly used non‐conducting mutant of *Shaker* (W434F), illustrating much greater tolerance of the W434F relative to either T439V or Y445F mutations, which both cause a dramatic loss of function even when expressed in a single channel subunit.^29^, ^30^, ^31^ This difference was attributed to the intersubunit nature of the T439 interaction. This previous finding of an essential structural role for this amino acid position motivated us to investigate the generality of this finding and the differing clinical outcomes in patients with mutations at this position in Kv7.3 in our study, versus previously described mutations at this position in Kv7.2 with severe clinical outcomes.^33^

The overall importance of position T313 (and its likely H‐bond interaction with Y319) is highlighted by the observation that Kv7.3[T313I] subunits cannot form functional homomeric channels, even using a high‐expressing Kv7.3[A315T] background channel (Figure 2). However, the effects of the T313I mutation in heteromeric channels (co‐expressed with Kv7.2) are much less pronounced (Figure 3). Modestly smaller current magnitudes are observed in the *Xenopus* oocyte system used for this study, and no significant differences in gating properties were observed between Kv7.2/Kv7.3[T313I] heteromeric channels and Kv7.2/Kv7.3 wild‐type channels (Figure 3). This observation is a departure from the powerful effects of disruption of intersubunit selectivity filter interactions in *Shaker* channels and likely highlights that other intersubunit interactions contribute significantly to stabilizing the selectivity filter of Kv7 channels.

Our findings also indicate variable outcomes of mutations at this position, depending on the amino acid substitution and mutated Kv7 subtype. For example, a Kv7.2 mutation at the analogous position (Kv7.2[T274M]) is linked to severe epileptic encephalopathy and global delay.^7^, ^33^, ^34^, ^35^ Functional characterization of this mutation in *Xenopus* oocytes results in a more pronounced effect on current suppression of heteromeric channels, relative to Kv7.3[T313I] (Figure 5).^33^ This difference in functional effects in heteromeric channels correlates with the severity of clinical outcomes. We investigated this difference in more detail by comparing equivalent mutations in both Kv7.2 and Kv7.3 and demonstrated non‐equivalent effects of these mutations in these different subtypes. Overall, mutations in Kv7.3 (T313I or T313M) were less severe than their comparators in Kv7.2 (Figure 5). In a broad context, this finding is consistent with reports that neonatal epilepsy (including severe epileptic encephalopathy) is far more commonly reported to arise from Kv7.2 mutations as opposed to Kv7.3 (Figure 1A).^7^, ^40^, ^46^ However, in terms of biophysical effects on channel function, there is not an obvious explanation for these diverging effects of Kv7.2 and Kv7.3. In addition to this apparent subtype‐dependent effect of certain mutations, other differences in physiological regulation of Kv7.2 and Kv7.3 likely contribute to the general differences in severity of diseases between these subtypes. For instance, Kv7.2 expression is more prominent than Kv7.3 in early development and infancy,^47^, ^48^ and these subtypes may contribute differently to channels with alternative stoichiometries (perhaps Kv7.2 homomeric channels, or other assemblies).^49^, ^50^, ^51^, ^52^ Another possibility is more penetrant effects of Kv7.2 versus Kv7.3 mutations on channel function may translate into stronger effects on structural and electrical remodeling of the axon initial segment, as has been observed in the presence of Kv7 inhibitors.^53^

Lastly, we hope to highlight the use of subtype‐selective Kv7 activators such as ICA‐069673 for investigating the assembly of heteromeric channels and inferring the functional impact of disease‐linked mutations in heteromeric channels. Since ICA‐069673 is strongly selective for Kv7.2 over Kv7.3 subunits, Kv7.2/Kv7.3 heteromeric channels have a distinct response from Kv7.2 homotetramers (Figure 4). This property reveals the impact of mutated versions of Kv7.3 on heteromeric channel function and clearly distinguishes homomeric Kv7.2 channels versus heteromeric assembly of Kv7.2/Kv7.3. Another useful approach used previously has been to exploit the differential sensitivity of Kv7.2 and Kv7.3 to extracellular tetraethylammonium.^54^, ^55^, ^56^ Our alternative approach using a more specific pharmacological agent is useful for resolution of distinct Kv7.2/Kv7.3 stoichiometries. Our findings also further validate prior reports of the stoichiometry‐dependent effects of ICA‐069673 and related compounds.

In summary, our study reports a novel variant in the Kv7.3 selectivity filter, linked to BFNE. The biophysical consequences of this mutation primarily involve suppression of function, without pronounced effects on voltage‐dependent gating. The assembly of functional channels comprising Kv7.2 and Kv7.3[T313I] subunits is unambiguously demonstrated based on sensitivity to a Kv7.2‐specific activator compound. Interestingly, the biophysical consequences of the Kv7.3[T313I] are less severe than reported in the prototypical *Shaker* potassium channel.

## CONFLICT OF INTEREST

None of the authors have any conflicts of interest to disclose. We confirm that we have read the Journal’s position on issues involved in ethical publication and affirm that this report is consistent with those guidelines.

## AUTHOR CONTRIBUTIONS

J. Maghera, J. Li, and S.M. Lamothe collected experimental data, performed molecular biology, and analyzed data. M. Braun, J.P. Appendino, and BPY Au collected clinical data and interacted with patients. All authors approved the final version of the manuscript.
